# Polyphenols and Tryptophan Metabolites Activate the Aryl Hydrocarbon Receptor in an in vitro Model of Colonic Fermentation

**DOI:** 10.1002/mnfr.201800722

**Published:** 2018-11-28

**Authors:** Jonna E. B. Koper, Linda M. P. Loonen, Jerry M. Wells, Antonio Dario Troise, Edoardo Capuano, Vincenzo Fogliano

**Affiliations:** ^1^ Department of Agrotechnology & Food Sciences Wageningen University Wageningen 6708 WG The Netherlands; ^2^ Department of Animal Sciences Wageningen University Wageningen 6708 WG The Netherlands; ^3^ Department of Agricultural Sciences University of Naples “Federico II” Portici 80055 Italy

**Keywords:** aryl hydrocarbon receptor, luteolin, microbiota, SHIME, tryptophan

## Abstract

**Scope:**

Many dietary phytochemicals have been reported to promote gut health. Specific dietary phytochemicals, such as luteolin, as well as specific microbial metabolites of tryptophan are ligands of the aryl hydrocarbon receptor (AhR), which plays a role in immunity and homeostasis of the gut barrier. Here, the fate of luteolin during colonic fermentation and the contribution of tryptophan metabolites to AhR activity in different parts of the colon are investigated.

**Methods and results:**

Several polyphenols are screened for AhR activation and oregano, containing the ligand luteolin, is added to batch cultures of human microbiota from the distal colon. Luteolin is rapidly metabolized, with no measurable increase in AhR activity. In the second experiment, using the Simulator of the Human Intestinal Microbial Ecosystem (SHIME), not all luteolin is metabolized in the ascending colon, but disappear rapidly in the transverse colon. The greatest AhR activity is due to microbiota‐derived metabolites of tryptophan, particularly in the descending colon.

**Conclusions:**

Luteolin in food is rapidly metabolized in the transverse colon. Tryptophan metabolism by the microbiota in the colon contributes substantially to the pool of lumen metabolites that can activate the AhR.

## 1 Introduction

The aryl hydrocarbon receptor (AhR) is best known for its role in detoxification of halogenated aromatic hydrocarbons such as polycyclic aromatic hydrocarbons (PAHs) and dioxins. Of these exogenous compounds, 2,3,7,8‐tetrachlorodibenzo‐p‐dioxin (TCDD) is the most potent activator and it induces diverse toxicological and biological effects.^1^, ^2^ Perspectives on the biological role of AhR have changed due to the establishment of a link between dietary or microbial agonists of the AhR and maintenance of host–microbe homeostasis in the intestine.^3^, ^4^, ^5^ Activation of AhR in the gut is essential for maintenance of intraepithelial lymphocytes (IELs) and IL‐22 producing innate lymphoid cells (ILC3s), which enhance the gut barrier functions and control the microbial load and composition.^5^, ^6^ Therefore, AhR is considered as a sensor that connects the outside environment with cellular processes with consequences for immune functioning.^5^, ^7^, ^8^

The AhR consists of a basic helix‐loop‐helix (bHLH) protein and belongs to the Per‐Arnt‐Sim (PAS) superfamily.^9^ The receptor is an intracellular, ligand‐activated transcription factor. In the cytosol, AhR forms a complex with two heat shock protein 90 molecules, an X‐associated protein 2, prostaglandin E synthase 3 (p23), AhR interacting protein (AIP), and AhR‐activated 9 (ARA9).^10^ Upon ligand binding to AhR, AIP is released leading to a conformational change, exposure of the nuclear location signal and translocation to the nucleus. In the nucleus, HSP90 is assumed to dissociate from the complex allowing interaction with ARNT and binding to the dioxin/xenobiotic response elements (DRE/XRE), leading to expression of AhR‐regulated genes.^11^

Certain dietary compounds like polyphenols, mainly flavonoids, and tryptophan derivatives have been reported as AhR ligands.^8^, ^9^, ^12^ Recent studies showed the major significance of dietary compounds like tryptophan and phytochemicals like indole‐derivatives in both intestinal and microbial homeostasis in relation to AhR in mouse colitis models.^13^, ^14^ As little is known about how metabolism of these compounds by microbes alters the AhR‐mediated signaling activity, we sought to study the kinetics of their release and the ability to activate the AhR using the Simulator of the Human Intestinal Microbial Ecosystem (SHIME), simulating the ascending (AC), transverse (TC), and descending (DC) colon.^15^ The aim of our research was to study the effects of dietary ligands on AhR and the effect of microbial fermentation of food matrix on the evolution of the formed ligands. Additionally, we investigated the relative contribution of microbiota‐derived tryptophan metabolites to the overall AhR activity in different parts of the colon.

## 2 Experimental Section

### 2.1 Chemicals

All chemicals used were purchased from Sigma–Aldrich (St. Louis, MO), unless stated otherwise. The tested polyphenols were: apigenin, baicalein, catechin, caffeic acid, 4‐*O*‐caffeoylquinic acid, chrysin, chlorogenic acid, curcumin (Indofine Chemicals, Hillsborough, NJ), daidzein, epicatechin, epigallocatechin gallate, formononetin, hesperetin, kaempferol, luteolin (Indofine Chemicals), myricetin, naringenin, quercetin, resveratrol (Indofine Chemicals), and rutin (Indofine Chemicals). Two different batches of dried oregano (*Origanum vulgare*) were purchased on the local marked and used for the batch and SHIME experiments.

### 2.2 Luteolin Extraction from Oregano

To determine the luteolin concentration in oregano, duplicate samples of 50 mg of milled dried oregano were mixed with 5 mL of 70% (v/v) methanol and sonicated for 60 min at 40 kHz, 100 W (HBM Machines B.V., Moordrecht, The Netherlands). The temperature during sonication ranged from 22 to ˂48 °C. After sonication, the extract was centrifuged at 1363 × *g* for 15 min and the supernatant was passed through a 0.20 µm cellulose filter (Phenomenex, Torrance, CA) and stored in the dark at room temperature.

### 2.3 Batch SHIME

SHIME (ProDigest, Belgium) was used to mimic the distal colon of three different donors. Microbial inoculum was first stabilized over a period of 2 weeks to adapt to the proximal and distal colon respectively as previously described.^16^ Fresh fecal samples were used to inoculate the TRIPLESHIME setup, consisting of a proximal and distal colon for three different donors. The two male and one female fecal stool donors were nonsmoking adults, between 25 and 35 years of age, with no prior history of antibiotic and probiotic use for at least 6 months and 3 weeks, respectively. For each donor, three double‐jacketed vessels were used, simulating one combined stomach/small intestine, a proximal (pH 5.6–5.9) and a distal colon (pH 6.6–6.9). Every 8 h, 70 mL fresh liquid feed (pH 2) entered the stomach vessel for each donor with a stable feed composition (1.2 g L^–1^ arabinogalactan, 2.0 g L^–1^ pectin, 0.5 g L^–1^ xylan, 0.4 g L^–1^ glucose, 3.0 g L^–1^ yeast extract, 1.0 g L^–1^ special peptone, 3.0 g L^–1^ mucin, 0.5 g L^–1^ l‐cysteine‐HCl, and 4.0 g L^–1^ starch^16^;). After 90 min, 30 mL of pancreatic juice (12.5 g L^–1^ NaHCO_3_; 6 g L^–1^ Oxgall, BD Biosciences, The Netherlands; 0.9 g L^–1^ pancreatin from porcine ≥3 * USP) was added. After 90 min of the small intestinal phase, the total volume was transferred to the proximal colon connected in series to the distal colon. The vessel volumes, pH, and retention times were kept constant at all times.^15^, ^17^

Freshly donated fecal sample was stored in a collection box with an anaerobic AnaeroGen bag (Oxoid, UK), at 4 °C for less than 8 h. A 20% (w/v) solution of the fecal sample was homogenized with phosphate buffer for 10 min using a Stomacher 400 circulator (Seward, UK). The sterilized phosphate buffer consisted of 8.8 g L^–1^ K_2_HPO_4_ (Merck KGaA, Germany), 6.8 g L^–1^ KH_2_PO_4_ (Merck KGaA) and 0.1 g sodium thioglycolate in demi‐water. The pH was adjusted to 7 and 15 mg sodium thionite (VWR, The Netherlands) was added before use. After mixing, the inoculum was centrifuged for 2 min at 500 × *g* and added in a concentration of 5 mL per 100 mL vessel volume. After the 2 weeks stabilization period, the microbiota was collected and stored with 50% sterilized cryoprotectant (a final concentration of 42% glycerol, 0.5 g L^–1^ cysteine HCl, 10 g L^–1^ trehalose and 3 g L^–1^ tryptic soy broth (Oxoid)) at –80 °C for further experiments.

The SHIME setup was modified to study oregano and luteolin fermentation in the distal colon. Three distal colon vessels were used, originating from the three different donors, inoculated with 4 mL of the frozen stabilized microbiota added to 400 mL feed per vessel. The microbiota was grown anaerobically overnight, with pH controlled in the range 6.6–6.9, followed by a 3‐day program with simulated feedings every 8 h. The experiment included three treatment days, with daily addition of 0.75 grams milled and sieved (<0.250 mm) dried oregano (Greek) per donor to the stomach phase. The concentration of luteolin in oregano was 6.4 ± 0.3 mg per 100 g. Samples were taken during fermentation and immediately centrifuged for 5 min at 9000 × *g* at a temperature of 4 °C. After centrifugation, the supernatants were filtered using a 0.20 µm cellulose filter and stored at –20 °C until further analyses. The three donors were used as biological replicates.

### 2.4 TWINSHIME

To simulate a complete microbial fermentation, including an ascending (pH 5.6–5.9, 250 mL), transverse (pH 6.15–6.4, 400 mL), and descending colon (pH 6.6–6.9, 300 mL), the TWINSHIME was used. Fecal inoculation was performed similarly to the batch SHIME experiment, including a 2‐week stabilization period. After stabilization, the microbiota suspension was frozen as described above. The experimental procedure consisted of addition of 1.5 g dried oregano per donor to the stomach phase, followed by a time series of sampling during fermentation at 10, 20, 30, 60, and 300 min in all colon parts. This batch of oregano contained 9.7 ± 0.3 mg luteolin per 100 gram oregano. Individual donors were used as biological duplicates.

### 2.5 AhR Activation

Luciferin transfected Dr Chemical Activated LUciferase gene eXpression (CALUX) reporter cells (BioDetection Systems, The Netherlands, mycoplasma free), HepG2 cells, and Caco‐2 cells were used to measure the AhR activation.

#### 2.5.1 Chemical Activated LUciferase gene eXpression

The reporter cells were grown in α‐MEM growth medium (Gibco, USA) with 1% penicillin/streptomycin (Gibco) and 10% heat inactivated fetal calf serum (Gibco), harvested by trypsin/EDTA and added to white clear bottom 96‐wells plate (Corning, USA) at a final concentration of 7.5 × 10^4^ cells per well. After 24 h incubation, the cells were stimulated with triplicate samples of the polyphenols listed in the chemicals section or controls and incubated for 24 h before performing the assay. All polyphenols and the positive control were dissolved in DMSO (Merck KGaA, Germany) and 1% final volume was added to the CALUX reporter cells to measure AhR activation. The microbial culture supernatant was added as 20% of final volume. After stimulation, the cells were washed twice with 200 µL per well PBS, lysed using 20 µL per well reporter lysis buffer (Promega, USA), followed by addition of 100 µL per well luciferase assay buffer (Promega). The luminescence was measured immediately after adding the assay buffer using a Spectramax M5 (Molecular devices, USA). The results of the AhR activation were expressed as a percentage of the activity obtained with the positive control consisting of 5 µm β‐naphthoflavone in DMSO.^18^ All results were corrected for the corresponding negative controls incubated with medium or DMSO (*n* = 3).

#### 2.5.2 CYP1A1 Gene Expression

The HepG2 cells (DSMZ, Germany) were grown in RPMI medium (Gibco) and Caco‐2 cells (ATCC, USA) in DMEM medium (Gibco), both with 1% penicillin/streptomycin and 10% heat inactivated fetal calf serum. The HepG2 cells were seeded in 12‐well plates (Corning) at a density of ≈7 × 10^5^ and used 24 h after seeding. The Caco2 cells were seeded at a density of 1.5 × 10^5^ in 12‐well plates and grown for 2 weeks with regular medium refreshments to allow for differentiation. The cells were stimulated with microbial culture supernatant in a 20% final volume concentration. After 6 h incubation, mRNA was extracted using a RNA isolation kit (Qiagen RNeasy mini kit, Germany) including an on‐column DNase treatment (Qiagen) and cDNA was synthesized of 1 µg mRNA (qScript, QuantaBio, USA). Quantitative PCR (qPCR) was performed using a Rotorgene machine (Qiagen) and SYBRgreen master mix (Promega, USA), to which 5 µL diluted (1:20) cDNA was added. The primers used were Cyp1A1 fw 5′GACCACAACCACCAAGAAC3′; rv 5′AGCGAAGAATAGGGATGAAG3′, GAPDH fw 5′TGCACCACCAACTGCTTAGC3′; rv 5′GGCATGGACTGTGGTCATGAG3′, and β‐actin fw 5′GGACTTCGAGCAAGAGATGG3′; rv 5′AGCACTGTGTTGGCGTACAG3′. The program used was 95 °C for 2 min, followed by 40 cycles of 95 °C for 3 s and 60 °C for 30 s. This was followed by a melt curve. The expression of Cyp1A1 was compared to both housekeeping genes and the results were similar following standardization to either gene. “‐RT” and non‐template controls were performed in every experiment and no amplification above background was detected. The results are shown as fold‐changes (2^–ΔΔCt^) calculated via the ΔΔCt method.^19^

### 2.6 Luteolin Analysis

Luteolin concentration was monitored according to Ferracane et al. with some modifications.^20^ Flavone was separated using an Ultimate 3000 U‐HPLC (Thermo Scientific, Bremen, Germany) equipped with a C18 column (XBridge, 100 × 2.1 mm, 2.6 µm Waters, UK) and a guard column of the same phase, both thermostated at 30 °C. The binary solvent system consisted of 0.1% v/v formic acid (solvent A) and 0.1% v/v formic acid acetonitrile (solvent B). The gradient elution was (min/%B): (0/20), (3/20), (7/80), and (9/80). The flow rate was 300 µL min^–1^ and the injection volume was 10 µL. The U‐HPLC was interfaced with a TSQ Quantum tandem mass spectrometer equipped with a heated electrospray source (HESI‐I, Thermo). Positive selected reaction monitoring mode with the following conditions was used: spray voltage 3000 V, sheath gas pressure 10 psi, auxiliary gas pressure 5 psi, and capillary temperature 260 °C. Luteolin was analyzed by using the mass transitions and collision energy (CE) given in parentheses, in bold the quantitative transition: (*m/z* [M+H]^+^ 287/89, CE: 42 V; **287/153**, CE: 31 V). Luteolin was quantified using a linear calibration curve with the external standard technique and the results were reported in ng mL^–1^.

### 2.7 Tryptophan Metabolites

Samples were centrifuged (21 700 × *g*, 10 min, 4 °C) and diluted five times in 0.1% formic acid and passed through a 0.22 µm cellulose filter (Phenomenex) before high resolution MS (HRMS) analysis. Chromatographic separation of tryptophan and tryptophan metabolites was achieved by using an Accela 1250 U‐HPLC (Thermo) equipped with a Luna Polar C18 column (50 × 2.1 mm, 1.6 µm, Phenomenex) and a guard column of the same phase, both at 40 °C. Mobile phases consisted of 0.1% v/v formic acid (A) and 0.1% v/v formic acid in acetonitrile (B) with the following gradient (min/%B): (0/2), (0.50/2), (9.5/70), and (12/70). The flow rate was 200 µL min^–1^, the column temperature was 40 °C and 5 µL was injected. The U‐HPLC system was interfaced to an Exactive Orbitrap HRMS (Thermo) and the analytes were detected through a heated electrospray interface (HESI‐II) in positive mode. The current ion of each analyte listed in Table S1 was scanned in the *m/z* range of 50–400. The resolving power was set to 75 000 full width at half maximum (FWHM, *m/z* 200) resulting in a scan time of 1 s. The interface parameters were: spray voltage 4.8 kV, capillary voltage 20.0 V, capillary temperature 295 °C, heater temperature 250 °C, and sheath gas flow and auxiliary gas flow were 30 and 9 arbitrary units, respectively. HRMS conditions were optimized by infusing a mixture of indole, indole‐3‐acetic acid, tryptophan, and l‐kynurenine (20 µg mL^–1^) at a flow rate of 3 µL min^–1^. Analyte concentrations were monitored by using the external standard technique while mass tolerance was set to 5 ppm. Three sets of calibration curves for tryptophan, indole, indole‐3‐propionic acid, indole‐3‐carboxyaldehyde, indole‐3‐acetic acid, l‐kynurenine, kynurenic acid, tryptamine, 3‐hydroxyanthranilic acid, anthranilic acid, and 6‐formylindolo(3,2‐*b*)carbazole (FICZ) were built in the range 0.455–911 ng mL^–1^. Intraday and interday assays were performed by monitoring three sets of calibration curves within the same day and in three different days. The slope among the calibration curves was compared to each replicate and the results were expressed as relative standard deviation RSD (%). Each sample was analyzed in triplicate and the concentrations given in nm. A summary of the analytical performances of the method is reported in the Supporting Information (Table S1), while the repeatability and reproducibility tests were always below 10% in the linearity range.

### 2.8 Microbial Analysis

16S sequencing of the V3‐V4 region was performed by BaseClear (Leiden, The Netherlands) and the results were analyzed using the CLC bio genomics workbench (Qiagen, The Netherlands), Microbial Genomics Toolbox. The SILVA 16S v128 99% database was used as reference database. Results show the relative abundances at phylum level for the three different donors.

### 2.9 Statistical Analysis

The statistical analyses were performed using GraphPad Prism 5 (GraphPad Software, La Jolla, CA). Results are shown as mean ± SEM). *p < 0.05, **p < 0.01, and ****p < 0.001 were considered statistical differences. Letters above bars represent classes of statistically significant different responses compared to each concentration. Each graph bar with the same letter is not statistically different. The AhR activation data and luteolin concentrations between the different fermentation time points were tested using a one‐way ANOVA followed by a Tukey post hoc analysis. A first order kinetics model was fitted to the luteolin degradation data in order to determine the rate constants for luteolin degradation on each of the three days using the Solver tool in Excel. Differences between days in fermentation rates were analyzed using a one‐way repeated measures ANOVA followed by a Tukey post‐hoc analysis. Differences between AC, TC and DC in AhR activation were tested using repeated measures two‐way ANOVA followed by a Bonferroni multiple comparisons test. The qPCR data was analyzed with ANOVA followed by a Tukey post hoc analysis using the ΔΔCt values. The heat map was calculated using OMICs (XLSTAT, Addinsoft, NY). Dendrograms were built by the agglomerative hierarchical clustering algorithm considering the concentrations (nm) of each tryptophan metabolite.^21^

## 3 Results and Discussion

### 3.1 Screening Dietary Phenolic Compounds for AhR Activation

**Figure 1:**
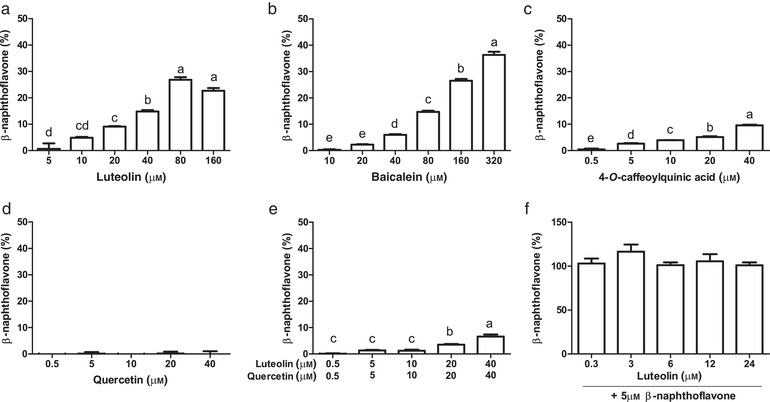
AhR activation measured with the CALUX reporter assay with luciferase production as readout, expressed as percent of the positive control (β‐naphthoflavone, 5 µm). a) Luteolin; b) Baicalein; c) 4‐*O*‐caffeoylquinic acid; d) Quercetin, e) Luteolin combined with an equivalent concentration of quercetin; f) Luteolin combined with 5 µm β‐naphthoflavone. Letters above the bar represent classes of statistically significant different responses compared to each concentration. Bars with the same letter are not statistically different.

Among all polyphenols listed in the chemical section tested for AhR activation, only luteolin, baicalein, and 4‐*O*‐caffeoylquinic acid showed a dose dependent AhR activation with an optimal concentration of 80, 320, and 40 µm, respectively (**Figure** 1). Interestingly, quercetin did not activate AhR, and actually reduced AhR activation by luteolin, suggesting an antagonistic effect of quercetin on AhR activation. When luteolin was combined with the ligand β‐naphthoflavone (Figure 1f), no differences in AhR activation were found, suggesting that luteolin does not show antagonistic effects on AhR when combined with another agonist. Chlorogenic acid also did not activate AhR, unlike its isomer 4‐*O*‐caffeoylquinic acid. Several studies have reported conflicting results on the capacity of polyphenols to activate AhR,^12^, ^22^, ^23^, ^24^, ^25^ most likely due to use of different cell types in the reporter assay.^26^ Baicalein has been reported to activate AhR using different cell lines, but quercetin is often reported as either a (weak) ligand or antagonist,^27^ consistent with our results (Figure 1e).

Luteolin and baicalein belong to a sub‐class of flavonoids named flavones. In their most stable conformation, flavone conformers can take on a planar structure, which is thermodynamically unfavorable to flavonols, because of the presence of a hydroxyl group at C3 position of the C carbon ring. Based on these observations and the fact that several known AhR ligands are planar (e.g., β‐naphthoflavone dioxin, FICZ), we speculate that a planar structure is an important structural feature of AhR ligands. Despite the fact that quercetin differs from luteolin by a single hydroxyl group, on C3 of the C ring, quercetin did not activate the AhR receptor in our assay. This is consistent with the fact that the most energetically stable conformer of quercetin is not a planar structure. Clearly, structural features other than the planar conformation are important for the activation of AhR because other flavones such as apigenin, which possesses a planar structure, did not activate AhR. The structure‐dependent AhR activation by flavonoids is also described by Jin et al., where they show that the number of hydroxyl groups plays an important role in AhR activity.^26^

Among the compounds that activate AhR, luteolin has dietary relevance as it is being found in several food sources such as oregano and parsley.^28^ Baicalein has been found in the roots of the *Scutellariae baicalensis*. This root is not consumed as a food, but frequently used in Chinese herbal medicine,^29^ while 4‐*O*‐caffeoylquinic acid is present in foods but its relative capacity to activate AhR is low. For these reasons, we decided to use oregano in our batch fermentation study because it is a rich source of luteolin.

### 3.2 Batch Fermentation of Oregano

**Figure 2:**
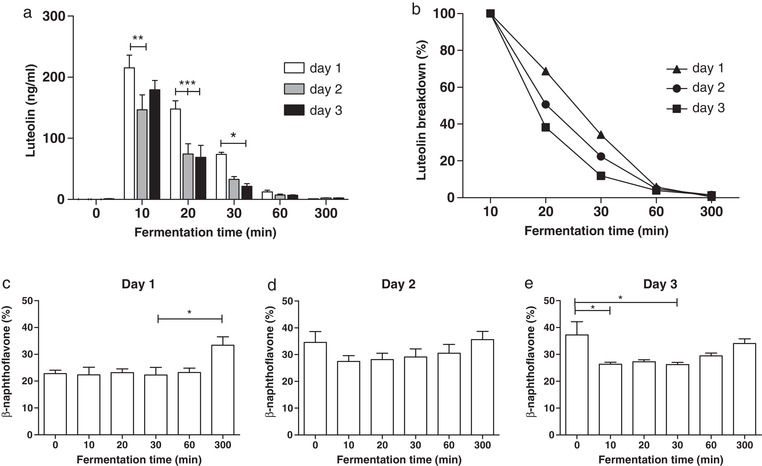
a) Luteolin concentration in distal colon supernatants over 300 min on three consecutive days of oregano feeding, *n* = 3 donors. b) Rate of luteolin breakdown as percentage of starting concentration on three consecutive days of oregano feeding, *n* = 3 donors. c–e) AhR activation in the distal colon on each day of consecutive oregano feeding, measured with the CALUX reporter assay using luciferase production as readout. Results are expressed as percent of the positive control (β‐naphthoflavone, 5 µm), *n* = 3 donors. Data are expressed as mean of three donors ± SEM, with *p < 0.05, **p < 0.01, and ***p < 0.001.

To study the metabolic fate of luteolin in oregano during distal colon fermentation, a batch fermentation was performed where 0.75 g of oregano per donor was added to the stomach phase of the digestion. The batch fermentation used in this experiment is a more physiological representation of human fermentation in vivo than conventional batch fermentation studies. After 10 min, the entire content of the small intestinal vessel was transferred directly to a vessel containing the microbiota of the distal colon. Results in **Figure** 2a indicated that, immediately after transfer, luteolin was rapidly metabolized by the microbiota of all donors. The luteolin bioaccessibility on day 1 was 179%, followed by 122% at day 2 and 149% at day 3. This showed that luteolin was readily and fully bio‐accessible from the finely grounded plant matrix that was used. After 60 min of fermentation, almost all luteolin was metabolized. Significant differences were observed in the rate of degradation of luteolin from oregano on different days (Figure 2b). On day 3, luteolin was more rapidly degraded than on day 1 (*p* < 0.05, **Table** 1), which may be due to a metabolic adaptation or a shift in the microbiota composition at the lower taxonomic levels, in response to daily addition of polyphenols. Labib et al. performed a batch fermentation of luteolin with pig microbiota, where luteolin was broken down and the single metabolite 3‐(3‐hydroxyphenyl)‐propionic acid was formed.^30^ Given its structural similarity to caffeic acid, there is no indication that the metabolite can activate AhR. In their study, the rate of luteolin degradation was lower than that measured in our human colon fermentation model. This might be due to differences in microbial density or composition, fermentation conditions or different concentrations of luteolin.^31^

**Table 1:** Rate constants (min^−1^) of luteolin degradation over three consecutive days of oregano feeding

	Day 1	Day 2	Day 3
Donor 1	0.0516	0.0785	0.1138
Donor 2	0.0413	0.0689	0.1236
Donor 3	0.0559	0.0695	0.0789

**Figure 3:**
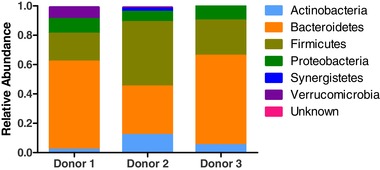
Microbiota composition at phylum level (relative abundance) for the three different donors used in the distal colon batch fermentation. Microbiota composition was determined after the 2‐week stabilization period in the SHIME and before addition of oregano.

Interestingly, a similar breakdown kinetics of luteolin and AhR activation capacity was found in all three donors so their microbial composition was compared. The microbial composition in the distal colons after the stabilization period of the three different donors is depicted in **Figure** 3. Donor 2 shows a different Firmicutes/Bacteroidetes ratio (1.33 compared to 0.32 and 0.39 for donor 1 and 3, respectively). Donor 2 also shows a higher relative abundance in Actinobacteria compared to donor 1 and 3. The relative abundance of Proteobacteria was similar for all donors. Differences of this magnitude are typical of the normal variation observed between individuals, including differences due to gender.^32^ In our study, the use of a standardized diet in the system throughout the stabilization period and experiments could have reduced the variability. However, even after a 2 weeks stabilization period with this diet, the microbial communities between donors preserve the original differences (data not shown).

Data of Figure 2c–e showed that supernatants from the colon fermentation have a significant capacity to activate AhR (between 20% and 30% of the reference). However, AhR activity does not change for up to 60 min after oregano addition, at which point the luteolin has been completely degraded (see Figure 2a), suggesting that at this concentration luteolin does not significantly contribute to the AhR activity present in the supernatants. Indeed, similar results were obtained with the microbial supernatant from the fermenter to which no oregano was added (time 0, Figure 2c–e), demonstrating that substantial amounts of AhR ligands were being produced by the fecal microbiota.

### 3.3 TWINSHIME Fermentation Oregano

To assess the metabolic fate of dietary luteolin in connection to AhR activation in the different parts of the lower gut, a SHIME fermentation study on oregano was performed. 1.5 g of oregano per donor was added to the stomach/small intestinal phase, after which the content of the stomach/small intestinal vessel was automatically transferred in series to the vessels simulating the AC, TC, and DC. The two different donors used in the SHIME study showed a different microbial composition (Figure S1, Supporting Information) with comparable functionality, similar to the previous experiment. Therefore, results are shown as biological duplicates.

**Figure 4:**
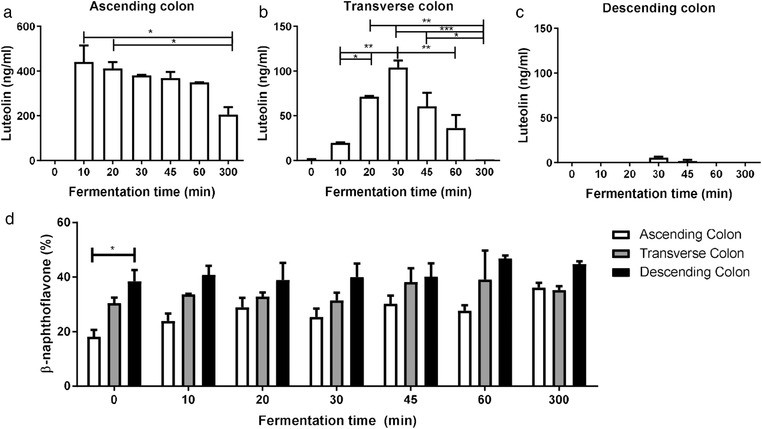
a–c) The luteolin concentration after feeding oregano to the SHIME system, presenting a partially‐dynamic system of ascending (a), transverse (b), and descending (c) colon connected in series. *n* = 2 donors. d) AhR activation measured with the CALUX reporter assay in the ascending, transverse, and descending colon during fermentation after addition of 1.5 g oregano per donor (*n* = 2 donors), using luciferase production as readout, expressed as percent of the positive control (β‐naphthoflavone, 5 µm). Data are expressed as mean of two donors ± SEM, with *p < 0.05, **p < 0.01, and ***p < 0.001.

**Figure** 4 shows the luteolin concentration over time in the AC (Figure 4a), TC (Figure 4b), and DC (Figure 4c). The most interesting finding was the limited metabolism of luteolin in the AC after 60 min (20.5%, *p* > 0.05), which was reduced to 53.3% after 300 min (*p* < 0.01). In contrast, a rapid decrease of luteolin was observed in the TC. The concentration of luteolin observed in the TC was initially lower than in the AC due to dilution upon transfer to the TC vessel, reaching a peak value of ≈100 ng mL^–1^ at 30 min, when the transfer was complete, after which it rapidly decreased. In the DC, low amounts of luteolin were present at 30 min due to its degradation in the TC and dilution upon transfer. Overall, these results suggest that breakdown of luteolin occurs primarily in the TC. The differential luteolin breakdown ability of the microbiota in the different part of the gut is a new finding that could have various biological implications. This evidence could be likely extended also to other polyphenols. In fact, the recent study of Wu et al. showed that absorption of polyphenols is influenced by location in the colon, with generally more absorption in the DC. Their data showed high recovery of polyphenols in the AC, whereas in the TC this is limited.^33^

**Figure 5:**
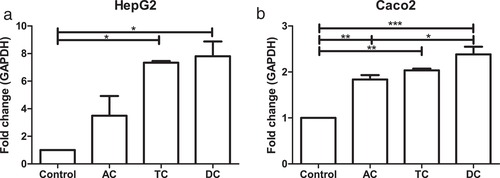
QPCR of Cyp1A1 gene expression as fold change compared to housekeeping gene GAPDH after stimulation of a) HepG2 and b) Caco‐2 cells with SHIME samples from ascending colon (AC), transverse colon (TC), and descending colon (DC) of donor 1 and 2. The experiment was repeated twice for both donors, with *p < 0.05, **p < 0.01, and ***p < 0.001.

Supernatants from each colon condition were tested for their capacity to activate AhR during the 300 min fermentation of oregano (Figure 4d). All supernatants strongly activated AhR with highest activity in the DC where no luteolin was present. These results are comparable to the qPCR data where the Cyp1A1 gene expression is higher in the DC compared to the TC and AC in both human liver (HepG2) and intestinal (Caco‐2) cells (**Figure** 5). It should be noted that the concentration of luteolin found in all the SHIME compartments is too low to account for the AhR signaling activity. The maximum recovered luteolin concentration was about 2 µm in the AC, which is about 20 times lower than what is needed to measure a significant AhR activation. The fermentation medium alone had no AhR activity and the luteolin released from oregano makes only a minor contribution to the total activity, indicating that the microbiota produces AhR activating ligands from other sources.

**Figure 6:**
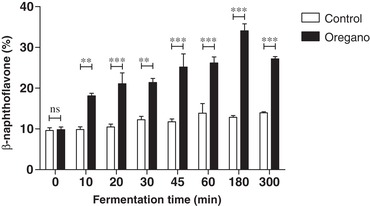
AhR activation in the ascending colon for a control fermentation compared to oregano fermentation, measured with the CALUX reporter assay using luciferase production as readout, expressed as percent of the positive control (β‐naphthoflavone, 5 µm), *n* = 2 donors. Data are expressed as mean of two donors ± SEM, with *p < 0.05, **p < 0.01, and ***p < 0.001.

**Figure** 6 shows the AhR activation in the AC. The basal activation is much lower than in the DC and a significant increase was observed after oregano feeding compared to a non‐oregano feeding control. Oregano induces AhR activation in the AC, which is comparable to the base level AhR activation in the DC (Figure 4d). These results suggested that food components contribute to the total amount of AhR agonists present in the proximal part of the colon but the vast majority of AhR ligands present in the TC and DC are produced by the microbiota. We can hypothesize that there is an optimal production of microbial‐derived AhR ligands along the colon and other AhR activators are needed when the microbial metabolites are not sufficiently present. This gap can be filled by dietary compounds.^34^ Which exact compounds trigger the AhR receptor in the AC in this case remains unknown, but luteolin alone cannot be responsible for this effect.

### 3.4 Role of Tryptophan Derivatives in AhR Activation

We hypothesized that a microbial source of AhR agonists in the SHIME supernatants were derived from the metabolism of tryptophan in the SHIME basal feed. The metabolism of tryptophan by certain gut bacteria has been shown to generate indole metabolites that exhibit both AhR agonistic and partial antagonistic activities.^8^, ^35^, ^36^, ^37^, ^38^ Furthermore, tryptophan supplementation in the diet of mice resulted in the production of tryptophan metabolites, which attenuated colitis in an AhR‐dependent fashion.^14^

**Figure 7:**
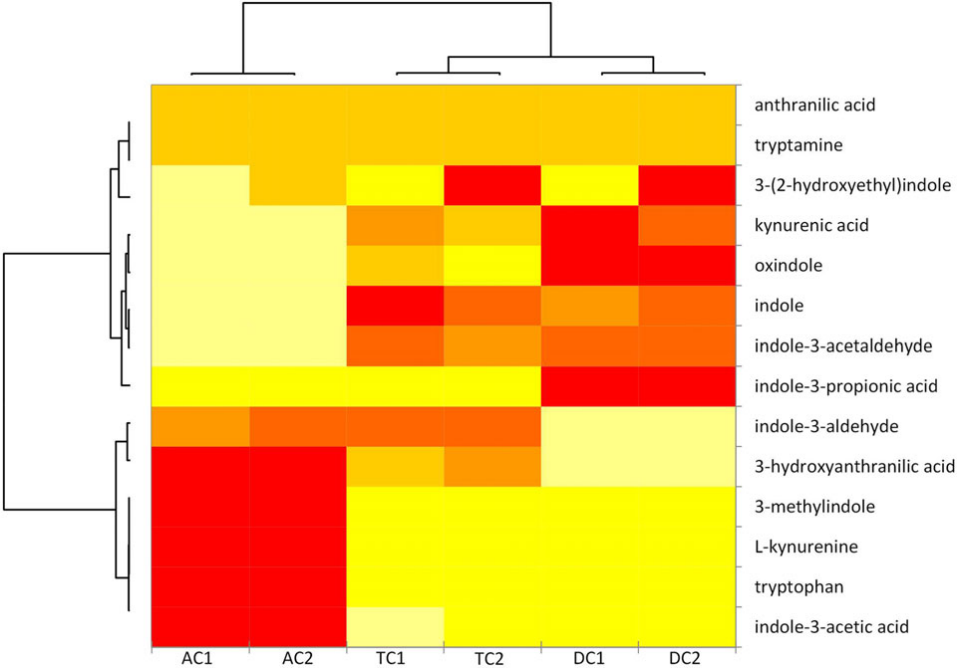
Heat map of tryptophan and tryptophan derivatives, from yellow (lower concentration) to red (higher concentration). Ascending colon (AC), transverse colon (TC), and descending colon (DC) of donor 1 and 2. The concentrations (nm) are reported in Figure S2, Supporting Information.

Results of measuring tryptophan metabolites (Table S1, Supporting Information) in fermentation supernatants are summarized in **Figure** 7 and their concentrations are given in Figure S2, Supporting Information. Ward's method was used to scale, center, and cluster data in order to depict hierarchical relationship between microbiota and the compounds formed.^21^ Colon sections of the two donors were grouped together as revealed by the horizontal dendrogram, confirming the similar metabolic capacity of microbiota of different donors. Metabolites were clustered into three main groups according to the concentration in the three different sections. Anthranilic acid, tryptamine, and 3‐(2‐hydroxyethyl)indole exhibited a constant concentration through the sections, while the other metabolites showed increased or decreased concentrations through the sections. In the bottom left corner of the dendrogram (AC1 AC2; colored red), the metabolites with highest concentration in the AC were tryptophan, l‐kynurenine, indole‐3‐aldehyde, 3‐hydroxyanthranilic acid, 3‐methylindole, and indole‐3‐acetic acid. However, these were measured in substantially lower amounts in the DC supernatants suggesting ongoing metabolism or degradation as they move through the colon. Oxindole, indole, kynurenic acid, indole‐3‐acetaldehyde, and indole‐3‐propionic acid were present at a higher concentration in the DC than in the AC supernatants. The tryptophan concentration changed from 3417 ± 100 nm in the AC, to a concentration of 372 ± 26 and 291 ± 0.9 nm in the TC and DC, respectively, while for instance indol‐3‐acetaldehyde increases from 3 ± 0.4 nm in the AC to 14 ± 0.0 and 16 ± 0.0 nm in the TC and DC, respectively. The tryptophan derivatives, kynurenine, tryptamine, indole‐3‐acetaldehyde, indole‐3‐acetic acid, and indole‐3‐aldehyde are known AhR ligands,^8^ explaining why the greatest AhR activation is found in the DC supernatants (Figure 4b).

An intrinsic limitation of the SHIME model system is that it does not model the intestinal absorption.^16^ As the intermediate tryptophan derivatives are not constantly removed as happens in vivo, an overestimation of AhR activity in the lumen of the human colon is expected because high levels of tryptophan are usually not reached in the distal colon. Besides lack of absorption, there is a discontinuous flow of liquids between colon sections in the system, resulting in a stricter cutoff between colon sections compared to in vivo where there is a continuous flow of material along the AC, TC, and DC.^39^

## 4 Concluding Remarks

Our results with the food component oregano demonstrated that AhR activity in the large intestine can be modulated by dietary compounds. Polyphenols with a planar structure were generally found to be good AhR ligands. Luteolin, an AhR agonist present in oregano, is unlikely to achieve significant AhR activation along the entire colon due to its rapid degradation. However, other dietary AhR ligands in oregano were shown to activate AhR in the ascending colon. Tryptophan metabolites generated by the microbiota are found in relatively high concentrations in all colon parts in our fermentation model, some of which are potent AhR ligands. Overall, it can be concluded that there is a dynamic formation of AhR ligands originating from dietary tryptophan by the gut microbiota and that other dietary AhR ligands can act as complementary AhR activators in the ascending colon.

## Conflict of Interest

The authors declare no conflict of interest.

## Supporting information

Supplementary
