# Supplementary material for: Polyphenols and Tryptophan Metabolites Activate the Aryl Hydrocarbon Receptor in an in vitro Model of Colonic Fermentation
Source: Mol Nutr Food Res. 2018 Nov 28;63(3):1800722. doi: 10.1002/mnfr.201800722 (PMC6588005; doi:10.1002/mnfr.201800722)

Supporting information

Table S1. HRMS performances. RT (retention time, min); TM (theoretical mass); EM (experimental mass, $[M+H]^+$); error (Δ ppm). The following compounds were quantified using chemical similarities toward the analytes in parenthesis (SciFinder, ACS 2018): 3-(2-hydroxyethyl)indole (indole-acetic acid), oxindole (indole), 3-methyl indole (indole), indole-3-acetaldehyde (indole-3-aldehyde).

**Compound Name**	**RT**	**Elemental Composition**	**TM**	**EM**	**Δ ppm**
L-kynurenine	2.3	C10H12N2O3	209.09207	209.09275	3.3
indole-3-acetaldehyde	2.6	C10H9NO	160.07569	160.07549	-1.2
3-hydroxyanthranilic acid	3.2	C7H7NO3	154.04987	154.04991	0.3
tryptophan	4.1	C11H12N2O2	205.09715	205.09754	1.9
3-methyl indole	4.3	C9H9N	132.08078	132.08028	-3.8
tryptamine	4.4	C10H12N2	161.10732	161.10719	-0.8
kynurenic acid	4.6	C10H7NO3	190.04987	190.04963	-1.3
anthranilic acid	4.9	C7H7NO2	138.05496	138.05490	-0.4
indole-3-aldehyde	5.5	C9H7NO	146.06004	146.05990	-1.0
indole-3-acetic acid	5.6	C10H9NO2	176.07061	176.07021	-2.3
3-(2-hydroxyethyl)indole	5.8	C10H11NO	162.09134	162.09155	1.3
indole-3-propionic acid	6.2	C11H11NO2	190.08626	190.08611	-0.8
indole	6.7	C8H7N	118.06513	118.06509	-0.3
oxindole	6.8	C8H7NO	134.06004	134.06022	1.3
6-formylindolo(3,2-*b*)carbazole	8.6	C19H12N2O	285.10224	285.10199	-0.9

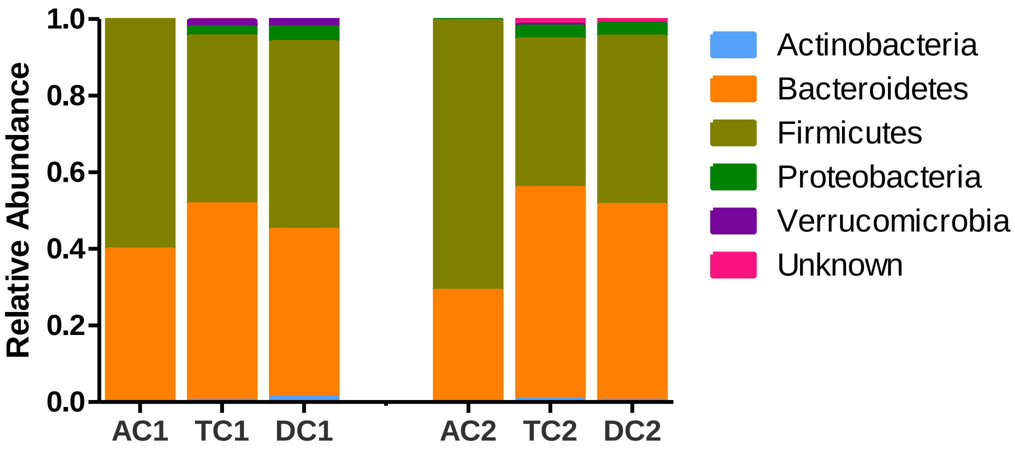

Figure S1. Relative abundance of microbiota used in the TWINSHIME® fermentation of 2 different donors before oregano treatment given in phylum level, analyzed by 16S sequencing. AC1/AC2 represents ascending colon donor 1/2; TC1/TC2 represents transverse colon donor 1/2; DC1/DC2 represents descending colon donor 1/2.

Figure S2. Concentration of tryptophan and tryptophan derivatives in ascending (AC), transverse (TC) and descending (DC) colon, n=2 donors.

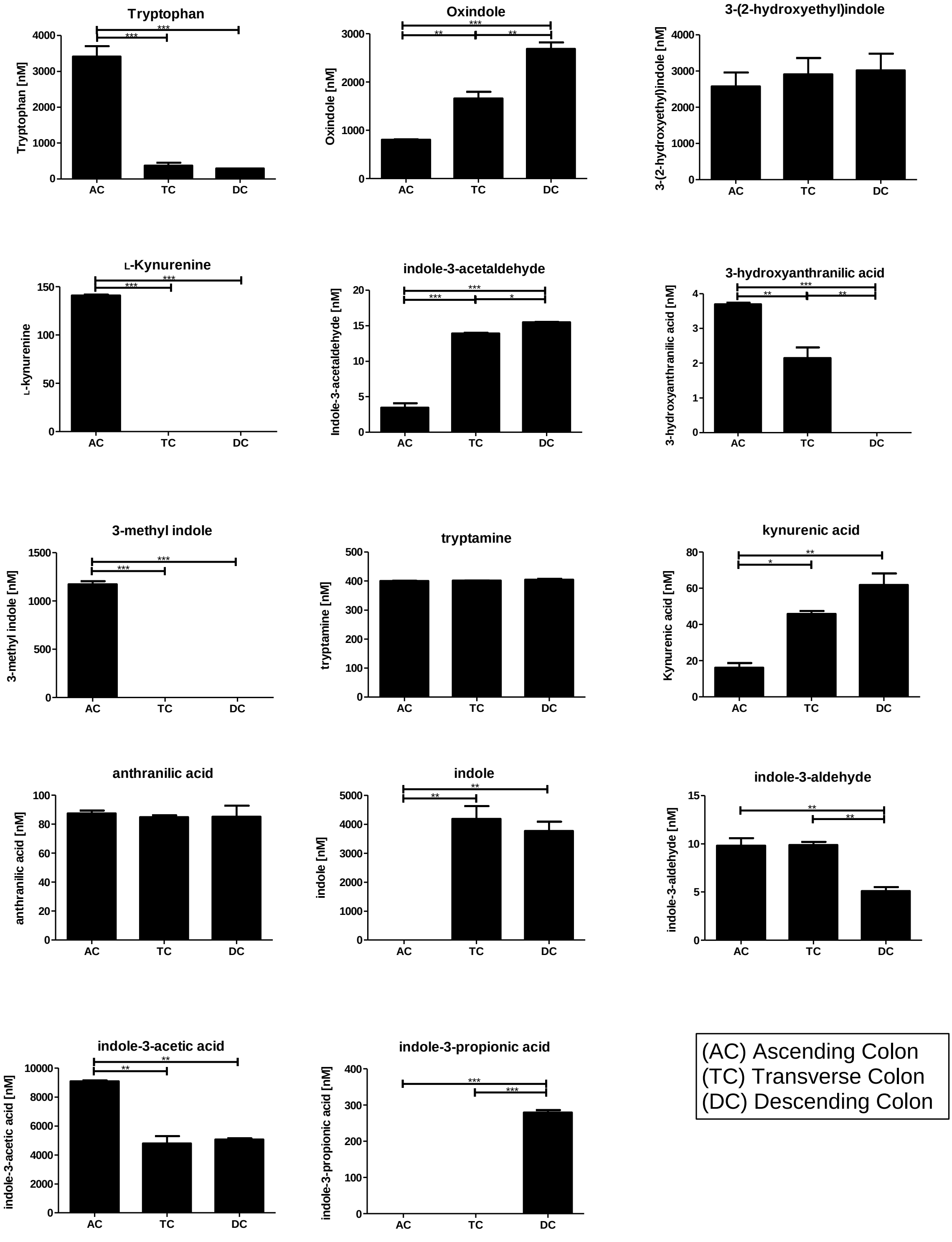

Supplement: Supplementary file 1 — Supplementary [file MNFR-63-na-s001.pdf]
